# The relationship between self-control and mental health problems among Chinese university students

**DOI:** 10.3389/fpubh.2023.1224427

**Published:** 2023-10-31

**Authors:** Mu He, Xiaoqing Zhan, Chang Liu, Ling Li, Xiaojie Zhao, Lei Ren, Kuiliang Li, Xi Luo

**Affiliations:** ^1^Chongqing Medical and Pharmaceutical College, Chongqing, China; ^2^Medical English Department, College of Basic Medicine, Army Medical University, Chongqing, China; ^3^Brain Park, Turner Institute for Brain and Mental Health and School of Psychological Sciences, Monash University, Clayton, VIC, Australia; ^4^College of General Education, Chongqing Water Resources and Electric Engineering College, Chongqing, China; ^5^Military Psychology Section, Logistics University of PAP, Tianjin, China; ^6^Military Mental Health Services and Research Center, Tianjin, China

**Keywords:** college students, impulse control, resisting temptation, mental health, network analysis, depression, anxiety

## Abstract

**Background:**

Mental health issues are often associated with poor self-control. Therefore, effective interventions against mental health problems should include self-control training. However, it is unclear whether the effect of self-control varies across different types of mental health problems.

**Methods:**

A cross-sectional survey was conducted using the convenience sampling method at five universities in Chongqing, China, where 1,409 students reported their demographic information, level of self-control, and symptoms of irritability, depression, and anxiety. Descriptive statistical methods and a network analysis approach were employed to explore the relationship between self-control and symptoms of irritability, depression, and anxiety among 1,409 students. The bridging links between self-control and the three mental health problems were analyzed.

**Results:**

The findings revealed a negative correlation between self-control and symptoms of irritability, depression, and anxiety among university students. Impulse control was found to be the bridge between self-control and irritability or anxiety symptoms, while resistance to temptation was the bridge between self-control and depressive symptoms.

**Conclusion:**

These results demonstrate the different relationship between self-control with irritability, anxiety, and depressive symptoms. The findings of this study may shed light on future mental health interventions for university students during potential public health emergencies, such as prior knowledge of the main types of psychological problems among university students, which may allow for the development of precise self-control intervention strategies, such as targeting impulsivity or resistance to temptation.

## 1. Introduction

The outbreak of COVID-19 has severely affected social activities and economic development (1). In response to the pronounced contagiousness exhibited by the virus during its initial phases, coupled with the grave manifestations and elevated fatality rate associated with the infection (2–4), China adopted stricter measures against the epidemic: a major nationwide blockade. As a result, university students had to change their lifestyle, such as switching offline classes to online classes, undergoing social isolation, and reducing outdoor activities (5, 6), which affected their physical and mental health (7). It was found that the mental health of university students deteriorated during the COVID-19 epidemic, with the prevalence of depression increasing from 23.8% (8) before the epidemic to 26.0% (9) and that of anxiety from 20.79% (10) to 25.0% (11). Therefore, it is important to investigate the mental health level of university students and find potential self-interventions.

Irritability is commonly associated with depression and anxiety (12). Irritability is considered a temporary emotional state characterized by impatience, intolerance, and uncontrollable anger (13), which may lead to verbal or behavioral outbursts that elicit internal or external aggression. However, emotional manifestations may not be observed, and irritability is subjectively unpleasant, which can be short or prolonged (14, 15). The impact of irritability on adults is often overlooked by researchers, and in the DSM-5, irritability is defined as a cross-diagnostic symptom, involving multiple psychiatric disorders such as depression and anxiety (16). Research on the relationship between irritability and anxiety and depression yielded inconsistent results (17), with network analysis results demonstrating a co-morbid relationship between irritability, anxiety, and depressive symptoms, as well as their separation relationship (18). In particular, university students are more likely to exhibit irritability symptoms when depressed or anxious (19). As a favorable predictor of future mental disorders (e.g., depression, anxiety) (20), irritability needs to be included in more empirical studies on the mental health of university students to promote their overall mental health level.

Research related to self-control has attracted increasing attention from researchers in recent years (21), often involving the integration of disciplines such as social and behavioral sciences. Self-control is considered to be an umbrella structure involving concepts of different disciplines, such as impulsivity, responsibility, self-regulation, executive function, and other cognitive domains that play an important role in adaptation to society (22). Self-control is reflected in different situations, such as holding back anger and showing patience with others or refusing to smoke or drink for physical health, in which people manage to reduce the internal struggle associated with self-control. For example, people tend to convince themselves that drinking a little alcohol on birthdays will not affect their health, which often makes self-control difficult (23). Self-control is often associated with mental health. Research has found that extreme tendencies to exert over- and under-control are associated with higher levels of depression, while anxiety symptoms increase with under-control (24). Levels of self-control can act as a more stable predictor of an individual’s mental health than depressive or anxiety symptoms that are more easily influenced by mood (25). Self-control is a protective factor in mental health (26), and according to the process model of ego depletion, performing self-control leads to a shift in motivation and attention, which increases positive emotional responses (27). Thus, self-control is a trainable and beneficial social competence (28). Considering the significant influence of self-control on mental health and the potential decline in self-control among university students during periods of isolation resulting from a lack of discipline (29), comprehending the interconnectedness between self-control and mental health within this demographic can effectively support the advancement of pertinent interventions.

As the mental health of university students during the COVID-19 epidemic is not promising, self-control training during isolation may need to be developed to help improve their mental health. Given irritability and anxiety symptoms play a more important role in the irritability, depression, and anxiety network compared with depressive symptoms (18), this study aimed to explore the bridging connections of self-control abilities with irritability, depression, and anxiety symptoms, respectively, through a network analysis approach. We hypothesized that (1) self-control is negatively correlated with irritability, depression, and anxiety symptoms; and (2) self-control is consistent with bridge symptoms in the irritability and anxiety networks while inconsistent in the depression network.

## 2. Methods

### 2.1. Participants

The present study utilized an online cross-sectional survey to collect data, which was conducted through the Questionnaire Star platform (www.wjx.cn) between 9 March 2020 and 12 March 2020. A convenience sampling method was adopted. The survey was uploaded onto the platform initially, and a QR code was, then, generated and disseminated to counselors from five universities and various social media platforms, including WeChat, Weibo, and QQ. The college counselors were asked to forward the survey QRs to the students in their charge, explain the purpose of the survey to the students, and require the students to voluntarily fill out the questionnaire. Only participants who were verified as university students were eligible to complete the survey. Before the survey, participants were informed about the purpose of the study and were required to provide their consent to participate. All survey questions were mandatory, and participants were randomly remunerated with 1–5 RMB for their participation. This study has been reviewed and approved by the Medical Ethics Committee of the Army Medical University (Project No. CWS20J007).

A total of 1,554 university students participated in the survey. Given the length of the questionnaire, which consisted of 48 items, participants were instructed to spend approximately 5 s on each question and the instructions, and a minimum completion time threshold of 300 s was set to ensure data accuracy. Consequently, after the exclusion of 145 participants who completed the survey in less than 300 s or were younger than 18 years (as minors in China) from the analysis, a final sample size of 1,409 participants was included. The validity of the survey was determined to be 90.67%.

### 2.2. Measurement tools

In this study, mental health problems among university students were assessed using the Irritability, Depression, and Anxiety Scale (IDA) developed by Snaith et al. (13). The IDA consists of four dimensions, namely, inward irritability, outward irritability, depression, and anxiety and includes 18 entries that are scored using a four-point Likert scale. The reliability and validity of the scale were previously established by Wilson et al. (30). In the current study, Cronbach’s α for the Chinese version of IDA was 0.846; McDonald’s ω was 0.852.

Based on the Self-Control Scale (SCS) developed by Tangney et al. (31), we used a revised Chinese version of the scale (32) to measure the self-control of the university students. The revised scale contained 19 questions that can be divided into five dimensions, namely, impulse control, healthy habits, resistance to temptation, work or study performance, and moderation from recreation. A five-point Likert scale was used, with 1 representing complete non-compliance and 5 representing complete compliance. The scale has good reliability, Cronbach’s α of the scale was 0.884, and McDonald’s ω was 0.886 in the current study.

### 2.3. Data analysis

In this study, mental health problems were categorized into three separate clusters including irritability, depression, and anxiety. The structure of irritability and self-control (IS), depression, and self-control (DS), and anxiety and self-control (AS) was estimated using the Graphical LASSO network method for undirected network estimation. Since the survey data were rank data, the networks were estimated based on Spearman rho correlation. To ensure that the networks were sparse and stable, the graphical Least Absolute Shrinkage and Selection Operator (LASSO) algorithm was used to eliminate spurious edges (33, 34). We set the Extended Bayesian Information Criterion (EBIC) hyperparameter γ at 0.5 to ensure the sensitivity and specificity of the network (33). The qgraph package was used to visualize three networks in which nodes represented dimensions of symptoms or self-control and edges represented regularized biased correlations between two nodes. Red edges indicate negative correlations, whereas blue edges indicate positive correlations and thicker edges indicate stronger relationships.

In the present study, the analysis of bridge nodes was conducted in two separate pre-defined communities, one including the five dimensions of self-control and the other including psychiatric symptoms (irritability symptoms, depressive symptoms, and anxiety symptoms). Bridge expected influence (BEI) values were calculated through the R package network tools (35) and were used to identify bridge nodes in both communities. The bridge expected influence referred to the sum of the edge weights of all nodes of a node connected to another community, and higher values of bridge expected influence indicated a higher likelihood of activation to the other community (35, 36).

The stability and accuracy of the network were calculated using the R package bootnet (37). The accuracy of each edge in the network was assessed by a 95% confidence interval (CI) of the edge weights (nboot = 2,000). Bridge expected influence stability was calculated by case-dropping bootstrap (nboot = 2,000) approach to calculate the correlation stability (CS) coefficient; CS coefficient represents the correlation between the original and subsample indices that remains at least 0.70 (95% of probability), and the maximum percentage of case data can be excluded. According to the recommendations, the ideal CS coefficient should be above 0.5 and not below 0.25 (37). The bootstrapped difference test was also used to determine the difference between edge and bridge nodes (38).

Finally, to assess the impact of gender as a potential confounding variable on the networks, we used the Network Comparison Test (39) to compare the gender variable in three networks. Comparison metrics included global strength and network structure differences (40).

## 3. Results

### 3.1. Descriptive statistics

In the present study, 1,409 university students were evaluated, of whom 326 (23.14%) were men. The participants’ ages ranged from 18 to 23 years, with a mean age of 20.14 ± 1.46 years (see Table 1). The total scores of all variables were significantly correlated (see Supplementary Table S1).

**Table 1:** The descriptive statistics of the participants (*N* = 1,409).

Variables	*n*(%)/*M* (SD)
**Gender**	
Male	326 (23.14)
Female	1,083 (76.86)
**Age (years)**	20.14 (1.46)
**Grade**	
Freshman	609 (43.22)
Sophomore	382 (27.11)
Junior	339 (24.06)
Senior	79 (5.61)
**Majors**	
Liberal arts	871 (61.82)
Science	538 (38.18)
**Internet at home**	
Yes	1,219 (86.52)
No	190 (13.58)

Age is the mean and standard deviation, the others are frequencies and ratios.

### 3.2. Irritability and self-control network analysis

**Figure 1:**
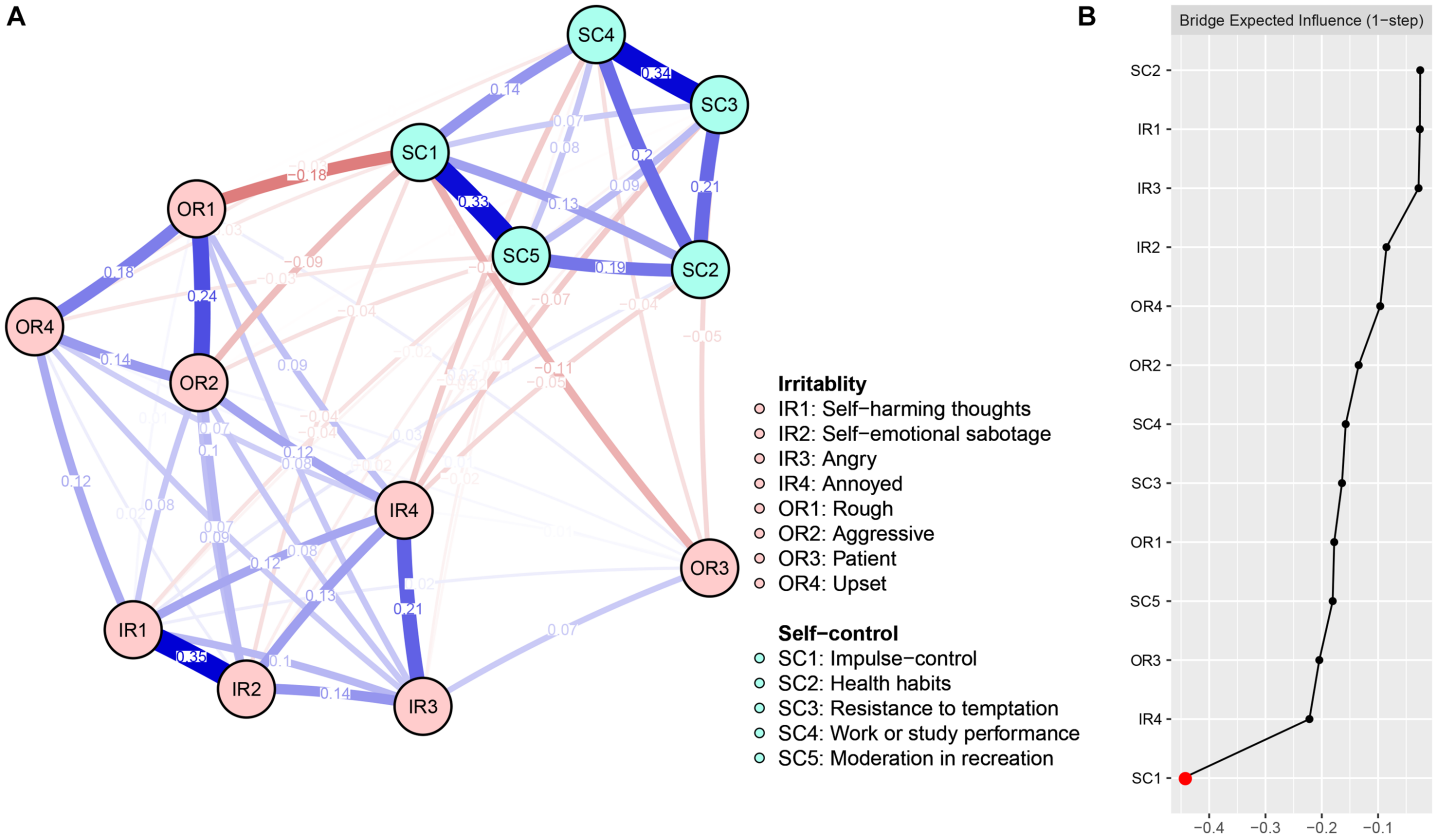
Network structure **(A)** and bridge expected influence **(B)** of IS among university students.

The network structures of IS are presented in Figure 1A. Out of the 78 edges with possible connections, 61 exhibited non-zero values (78.21%). The edges that demonstrated the strongest connections within the IS network were IR1-IR2 (weight = 0.35), F3-F4 (weight = 0.34), and F1-F5 (weight = 0.33), ranking in descending order. With the exception of SC2-IR1, which displayed a positive correlation (weight = 0.03), all other linked edges exhibited a negative correlation (weight ranged from 0.00018 to 0.18) in both communities. The relatively narrow bootstrapped 95% confidence interval provided credibility to the accuracy of the IS network edges (Supplementary Figure S1). The present network edge weight CS coefficient achieved 0.75, exceeding the recommended value of 0.5 (37). Supplementary Figure S2 illustrates the bootstrapped difference test for edge weights.

Figure 1B shows the centrality of the IS network bridge expected influence. Among the IS network, Node SC1 (BEI = −0.44) demonstrated the lowest bridge expected influence value. The correlation stability coefficient of 0.75 for the bridge expected influence is presented in Supplementary Figure S3, indicating an acceptable level of stability. The bootstrapped difference test for the node’s bridge expected influence is displayed in Supplementary Figure S4.

### 3.3. Depression and self-control network analysis

**Figure 2:**
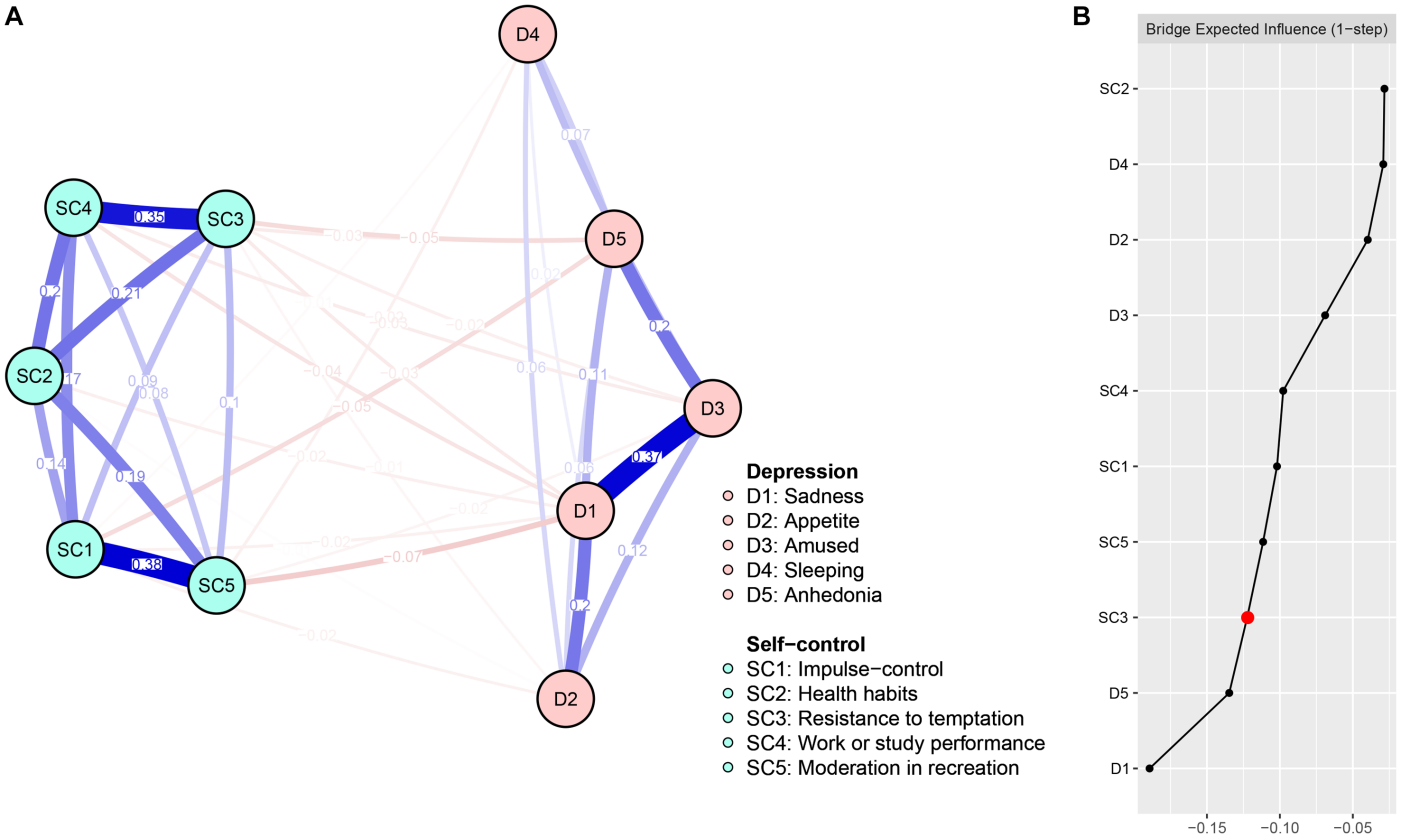
Network structure **(A)** and bridge expected influence **(B)** of DS among university students.

The network structures of DS are illustrated in Figure 2A, showing that among the 45 edges connecting the nodes, 80% (36) were non-zero. The DS network displayed the highest strengths for edges F1-F5 (weight = 0.38), D1-D3 (weight = 0.37), and F3-F4 (weight = 0.35), ranging in descending order. The association between all dimensions of self-control and depressive symptoms was negative (weight range: 0.005–0.073). The DS network edges were accurate, with relatively narrow bootstrapped 95% CI (Supplementary Figure S5). The current network edge weight CS coefficient was 0.75, surpassing the recommended value of 0.5 (37). The bootstrapped difference test for edge weights is displayed in Supplementary Figure S6.

Figure 2B shows the centrality of the DS network bridge expected influence. Node SC3 (BEI = −0.22) exhibited the lowest bridge expected influence among nodes in the self-control community. The correlation stability coefficient of the bridge expected influence was 0.44 (refer to Supplementary Figure S7), indicating that the bridge expected influence was acceptably stable. The bootstrapped difference test for node bridge expected influence is presented in Supplementary Figure S8.

### 3.4. Anxiety and self-control network analysis

**Figure 3:**
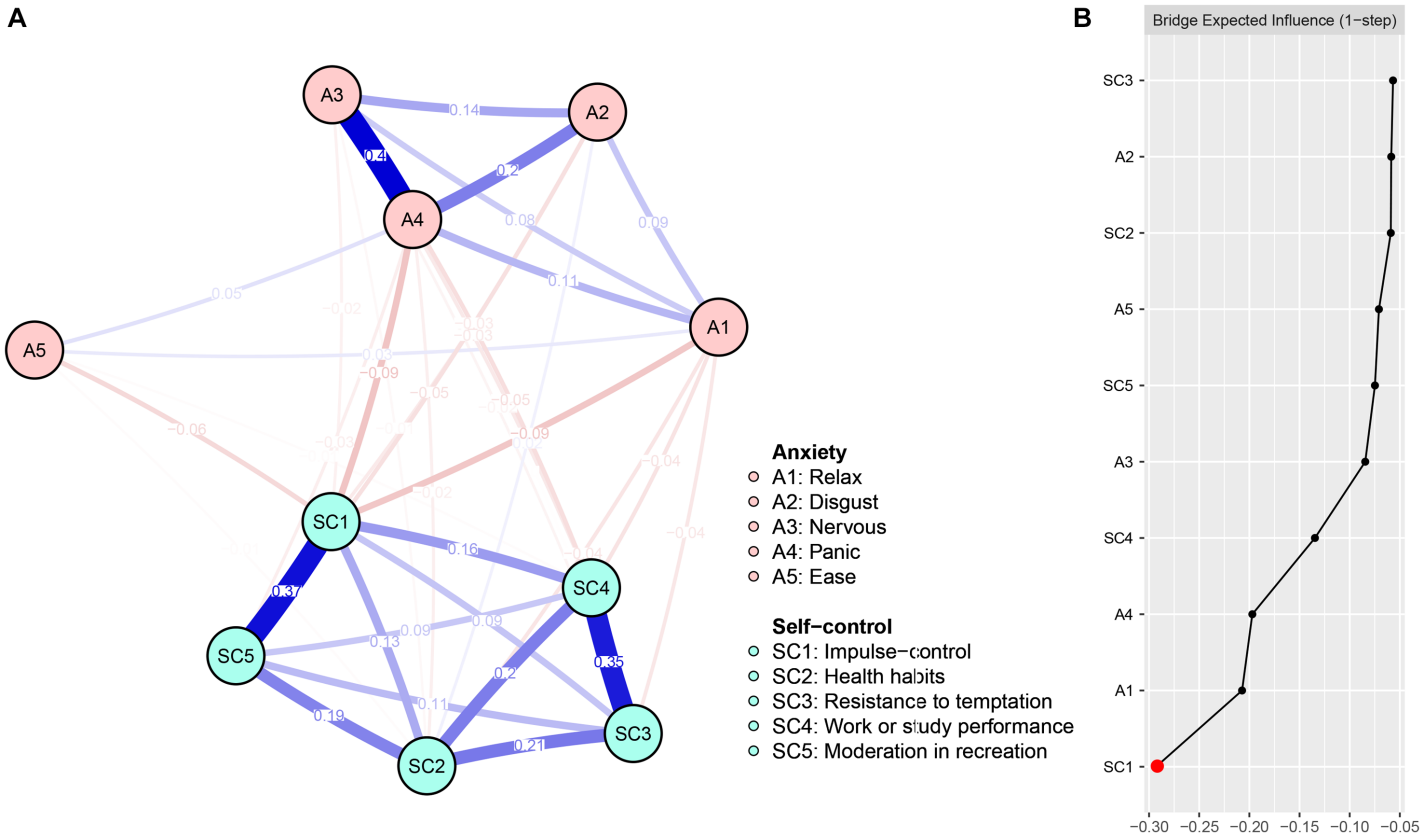
Network structure **(A)** and bridge expected influence **(B)** of AS among university students.

The network structures of AS are presented in Figure 3A, with 37 out of 45 possible edges (82.22%) being non-zero. The edges with the highest connections in the AS network were A3-A4 (weight = 0.40), F1-F5 (weight = 0.37), and F3-F4 (weight = 0.35), ranking in descending order. The association between all dimensions of self-control and anxiety symptoms was found to be negative, with the weight ranging from 0.006 to 0.092. Additionally, the AS network edges were accurate, with relatively narrow bootstrapped 95% confidence intervals (refer to Supplementary Figure S9). The current network edge weight CS coefficient was 0.75, exceeding the recommended value of 0.5 (37). The bootstrapped difference test for edge weights is illustrated in Supplementary Figure S10.

Figure 3B displays the centrality of the AS network bridge expected influence. Among the nodes in the AS network, SC1 (BEI = −0.29) exhibited the lowest expected influence. The stability coefficient for the correlation of the bridge expected influence was 0.60 (refer to Supplementary Figure S11), indicating that the bridge expected influence was adequately stable. The bootstrapped difference test for node bridge expected influence is presented in Supplementary Figure S12.

The results of the comparative network analyses showed that there were no significant differences regarding gender variables among the three networks (irritability and self-control network: *M* = 0.16, *p* > 0.05; *S* = 0.03, *p* > 0.05; depression and self-control network: *M* = 0.11, *p* > 0.05; *S* = 0.13, *p* > 0.05; and anxiety and self-control network: *M* = 0.13, *p* > 0.05; *S* = 0.28, *p* > 0.05). The results suggest that the gender variable is not a potential confounding variable influencing the network.

## 4. Discussion

The quarantine during the COVID-19 epidemic forced a rapid shift in the way university students live and study (41), leading to social isolation, increased anxiety, and depression (42). Self-control may be compromised because of campus closures, which resulted in a shift from offline to online courses, a change in study locations from classroom to home, and a lack of proper supervision. The current study investigated the interrelationship between self-control and symptoms of irritability, depression, and anxiety among university students during COVID-19 isolation. In general, self-control is related to irritability and anxiety symptoms in a similar way while related to depressive symptoms in a different way, and attention should be paid to the identification of these relationships when conducting interventions.

In the three networks constructed, the correlations of self-control with irritability, depression, and anxiety symptoms were overall negative, except for a positive correlation of SC2 (healthy habits)-IR1 (self-harm thoughts). These results are generally consistent with those of the previous research in which self-control is negatively correlated with depression and anxiety (43, 44). The findings support self-control training as a potential way to intervene in psychological problems. There are two potential explanations for the observed positive correlation between SC2 and IR1. On the one hand, COVID-19 isolation disrupts pre-existing habits, affecting adherence to healthy habits, such as getting up early, and leads to the development of bad habits, such as staying in bed during home isolation (45, 46). On the other hand, decreased self-control increases unhealthy activities of university students, such as internet or smartphone addiction, reduced study motivation, and other behaviors that can easily lead to guilt and self-harm thoughts. These two aspects become unstable during COVID-19 isolation, which may blur the SC2-IR1 relationship, based on a weaker marginal weight (weight = 0.03) that may support this idea. However, further studies in different scenarios are still needed to determine the real relationship.

The bridge linkage analysis showed that node SC1 (impulse control) was connected to irritability and anxiety symptoms as the bridge node with the strongest self-control, supporting our first study hypothesis. Notably, many studies have shown that irritability predicts depression and generalized anxiety disorder (47–49). Our results reveal that irritability is similar to anxiety symptoms rather than depressive symptoms at a symptom level. In the DSM-5, irritability is described as a symptom of generalized anxiety disorder, and persistent irritability is present in 90% of patients with generalized anxiety disorder seeking treatment (50). Studies have shown that abnormalities in the amygdala-prefrontal-parietal circuitry in young adults underlie the neurological coexistence of anxiety and irritability (51). This evidence indicates a greater resemblance in the clinical manifestation and physiological attributes of anxiety and irritability. However, it should be noted that the current study investigated depressive symptoms as generalized depressive symptoms rather than distinguishing subtypes of depressive symptoms, such as manic depression or disruptive mood dysregulation disorder that was shown to be significantly associated with irritability (52). Future research is needed to further confirm the relationship between provocation and different subtypes of depression.

Impulse control disorder (ICD), which usually manifests as difficulty controlling emotions and behaviors (53), is prevalent among university students and has a high prevalence (10.4%) (54). The study has demonstrated a significant correlation between impulse control disorders and irritability. Irritability, a multifaceted issue, is regarded as a physiologically induced emotional complex marked by heightened sensitivity to sensory stimuli and a simultaneous reduction in thresholds, not primarily influenced by cognitive processes. This condition frequently manifests as angry or aggressive responses to stimuli that are typically less bothersome and is directly linked to physiological or biological factors such as hunger, sleep deprivation, and chronic physical isolation (55). Previous research has found that emotion-driven impulsive control plays a potential role in reducing aggression, particularly aggression that exhibits angry and impulsive responses to perceived acute stress (56, 57). Given the comorbidity of irritability and anxiety (58), impulse control may similarly reduce anxiety levels. Research suggests that greater effort of control is associated with lower anxiety, possibly due to the ability of greater control effort to distract the anxious person from “slowing down” and objectively interpreting internal experiences or suppressing inaccurate thoughts about anxiety-related feelings (59). This evidence suggests that impulse control may be one way to reduce irritability and anxiety symptoms and that interventions of cognitive and behavioral training (CBT) (60) and self-control training (SCT) (61) may be considered for coping with irritability and anxiety in university students.

One view is that trait self-control is not usually achieved through efforts to resist temptation but rather a healthy habit that keeps individuals less tempted from straying away from their goals (62). Research suggests that when self-control is low, people are more likely to develop unhealthy behaviors such as smoking, excessive alcohol consumption (63), and internet addiction (64). In contrast, our study suggests that self-control is primarily linked to depressive symptoms through resisting temptation (a bridge node between self-control and depressive symptoms) rather than healthy habits, and the degree and type of temptation may affect this linkage (65). Taken together, these findings may explain the negative correlation between self-control and depressive symptoms. The mechanism underlying this correlation is that more exposure to temptations because of bad habits makes individuals fail to fulfill their goals, leading to depressive symptoms. For example, university students suffer from sleep problems due to internet addiction, causing depressive symptoms (66–68). Therefore, we emphasize the role of resistance to temptation in depression and found the strongest negative correlation between resistance to temptation and anhedonia. Many temptations may lead to addiction, such as the temptation of money leading to gambling addiction, the temptation of surfing online leading to internet addiction, and the temptation of alcohol leading to alcohol addiction. These addictive behaviors will cause euphoria in individuals, and withdrawal from addiction will lead to anhedonia (69). Therefore, we suggest that university students should first develop good habits, such as staying away from tempting stimuli such as alcohol and tobacco, and trying to resist temptation when faced with it. In addition, studies have shown that positive emotions can increase resistance to temptation when temptation activates long-term health goals (70). On the one hand, positive emotions can promote resistance to temptation; on the other hand, positive emotions can directly alleviate depressive symptoms. The above results suggest that developing positive emotion regulatory preferences in university students (71) may have an enhancing effect on resistance to temptation and depressive symptoms.

There are two limitations in this current study. First, the data were collected from university students isolated during the COVID-19 epidemic. As isolation may affect the self-control of university students, it is unclear whether the findings can be generalized to the current non-isolated university students, and future research should examine the relationship between self-control and psychological symptoms among non-isolated university students. In addition, as the university students were selected as participants through convenience sampling methods, the results should be interpreted with caution when generalized to other groups. Future studies should include adolescents and adults. Moreover, this study adopted a cross-sectional survey, and we did not use a double sample or conduct experimental validation of the bridge node. Future research should consider the use of longitudinal research design and multi-sample analysis or the use of experimental validation of the effectiveness of the core objectives of the intervention.

## 5. Conclusion

Our research sheds light on the complex relationship between self-control and mental health problems by demonstrating that controlling impulses is a protective factor against irritability and anxiety symptoms, and resisting temptation is a protective factor against depressive symptoms. Our findings suggest that self-control interventions among university students isolated during public health emergencies could target impulse control (for irritability and anxiety symptoms) and resisting temptation (for depressive symptoms). For future intervention, the types of mental health problems among university students need to be assessed first to determine the targeted intervention goals. In conclusion, future research should focus on the effectiveness of self-control as a target for intervention, such as the effectiveness of self-control training (working memory training or episodic future thinking) in reducing mental health problems among university students.

## Data availability statement

The original contributions presented in the study are included in the article/Supplementary material, further inquiries can be directed to the corresponding authors.

## Ethics statement

The studies involving humans were approved by Medical Ethics Committee of the Army Medical University. The studies were conducted in accordance with the local legislation and institutional requirements. The participants provided their written informed consent to participate in this study.

## Author contributions

MH, XZ, KL, and XL contributed to the conception and design of the study. XJ and LL organized the database. LR and KL performed the statistical analysis. MH, XZ, and KL wrote the first draft of the manuscript. CL, LR, KL, and XL wrote sections of the manuscript. All authors contributed to the article and approved the submitted version.
