# Supplementary material for: The relationship between self-control and mental health problems among Chinese university students
Source: Front Public Health. 2023 Oct 31;11:1224427. doi: 10.3389/fpubh.2023.1224427 (PMC10644003; doi:10.3389/fpubh.2023.1224427)
Supplement: Supplementary file 1 [file Data_Sheet_1.docx]

**Supplementary Materials**

1. Table S1. Pearson correlation coefficient of the variables

**The results of IS network**

1. Figure S1. Accuracy of edge weights
2. Figure S2. Bootstrapped difference test for edge weights
3. Figure S3. Stability of node bridge expected influences
4. Figure S4. Bootstrapped difference test for node bridge expected influences

**The results of DS network**

1. Figure S5. Accuracy of edge weights
2. Figure S6. Bootstrapped difference test for edge weights
3. Figure S7. Stability of node bridge expected influences
4. Figure S8. Bootstrapped difference test for node bridge expected influences

**The results of AS network**

1. Figure S9. Accuracy of edge weights
2. Figure S10. Bootstrapped difference test for edge weights
3. Figure S11. Stability of node bridge expected influences
4. Figure S12. Bootstrapped difference test for node bridge expected influences

Table S1. Pearson correlation coefficient of the variables

|  | **Depression** | **Anxiety** | **IR** | **OR** | **S1** | **S2** | **S3** | **S4** | **S5** |
| --- | --- | --- | --- | --- | --- | --- | --- | --- | --- |
| **Depression** | 1 |  |  |  |  |  |  |  |  |
| **Anxiety** | 0.512 | 1 |  |  |  |  |  |  |  |
| **IR** | 0.458 | 0.513 | 1 |  |  |  |  |  |  |
| **OR** | 0.366 | 0.437 | 0.610 | 1 |  |  |  |  |  |
| **S1** | -0.277 | -0.377 | -0.396 | 0.518 | 1 |  |  |  |  |
| **S2** | -0.216 | -0.270 | -0.249 | -0.291 | 0.503 | 1 |  |  |  |
| **S3** | -0.273 | -0.270 | -0.331 | -0.350 | 0.479 | 0.531 | 1 |  |  |
| **S4** | -0.270 | -0.313 | -0.317 | -0.370 | 0.515 | 0.542 | 0.617 | 1 |  |
| **S5** | -0.267 | -0.280 | -0.371 | -0.409 | 0.607 | 0.511 | 0.461 | 0.461 | 1 |

**The results of IS network**


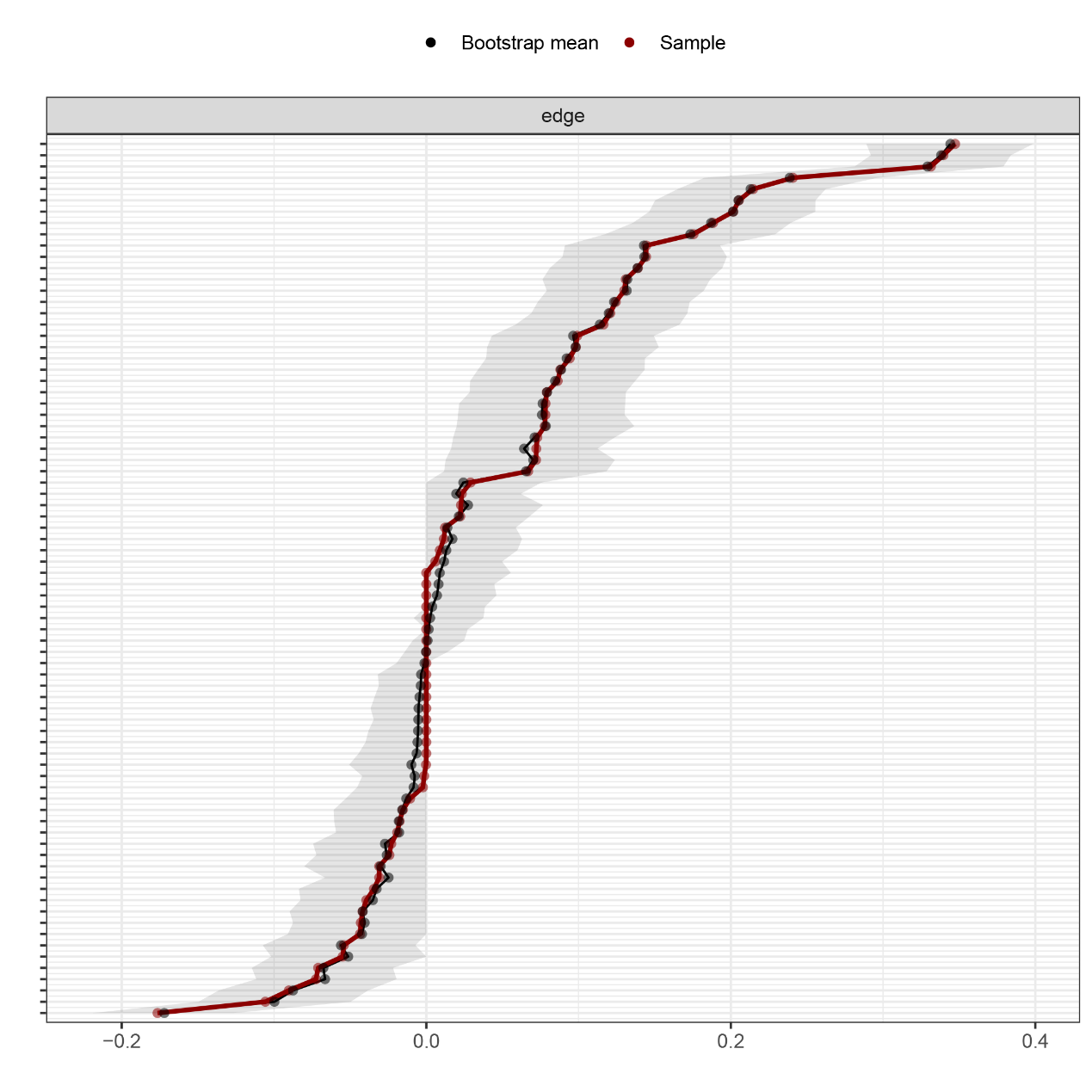


Figure S1. Accuracy of edge weights

*Note*: The red line depicts the sample edge weights and the gray bar depicts the bootstrapped confidence interval.


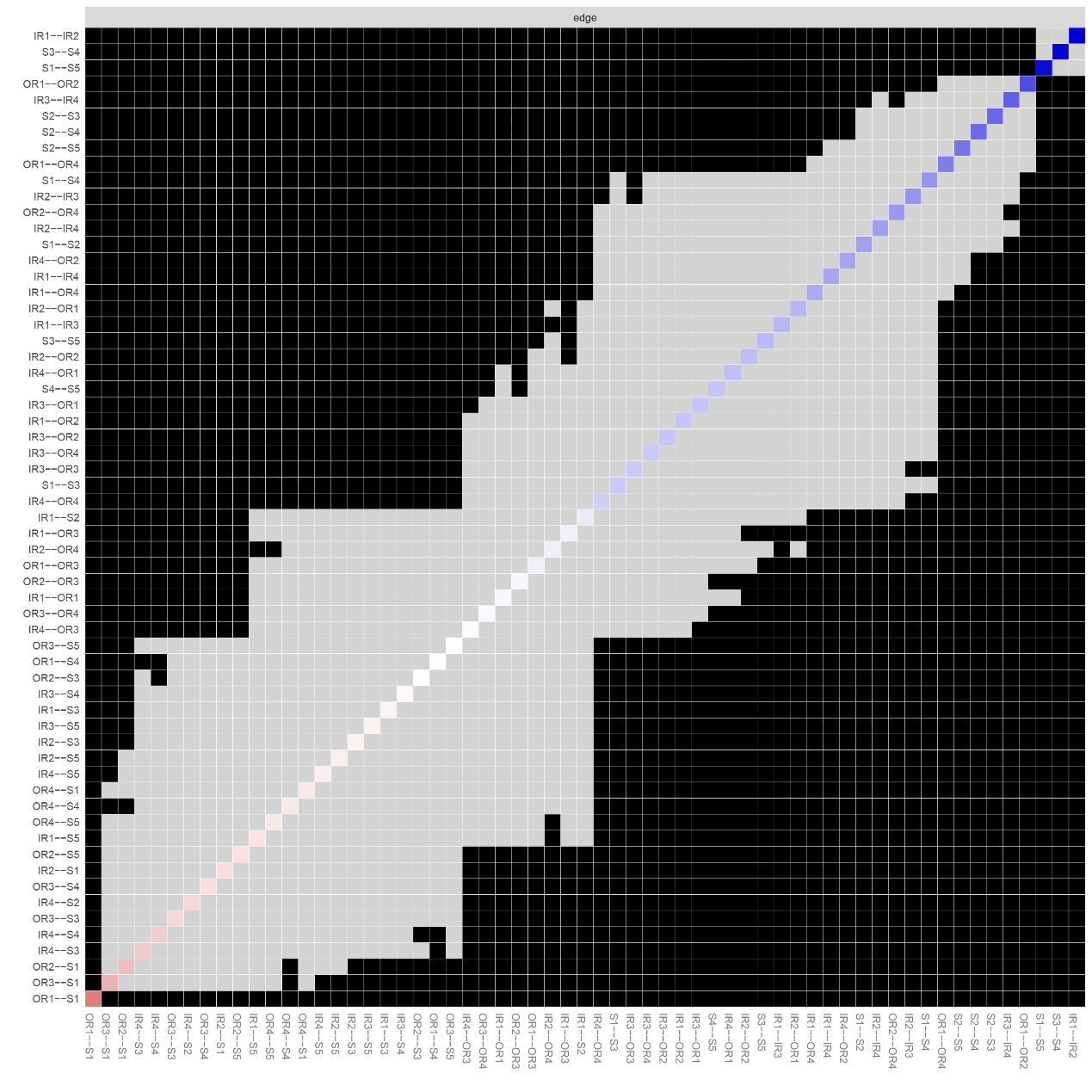


Figure S2. Bootstrapped difference test for edge weights

*Note*: Gray boxes indicate edge weights that do not differ significantly from one another, while black boxes indicate edge weights that do differ significantly. Blue and red boxes on the diagonal correspond to edge weights with positive and negative correlations, respectively.


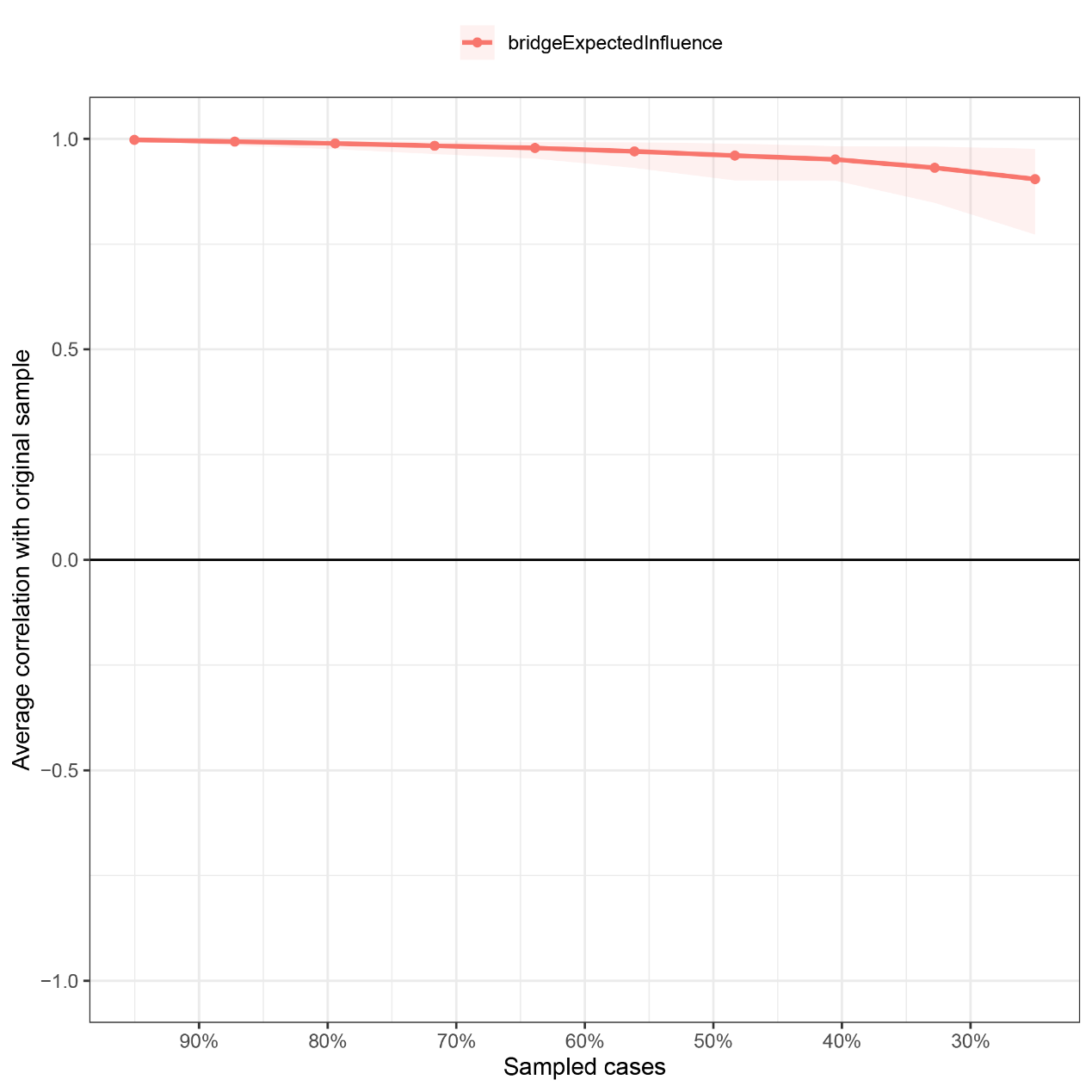


Figure S3. Stability of node bridge expected influences

*Note*: The red bar represents the average correlation between node bridge expected influences in the full sample and subsample with the red area depicting the 2.5th quantile to the 97.5th quantile.


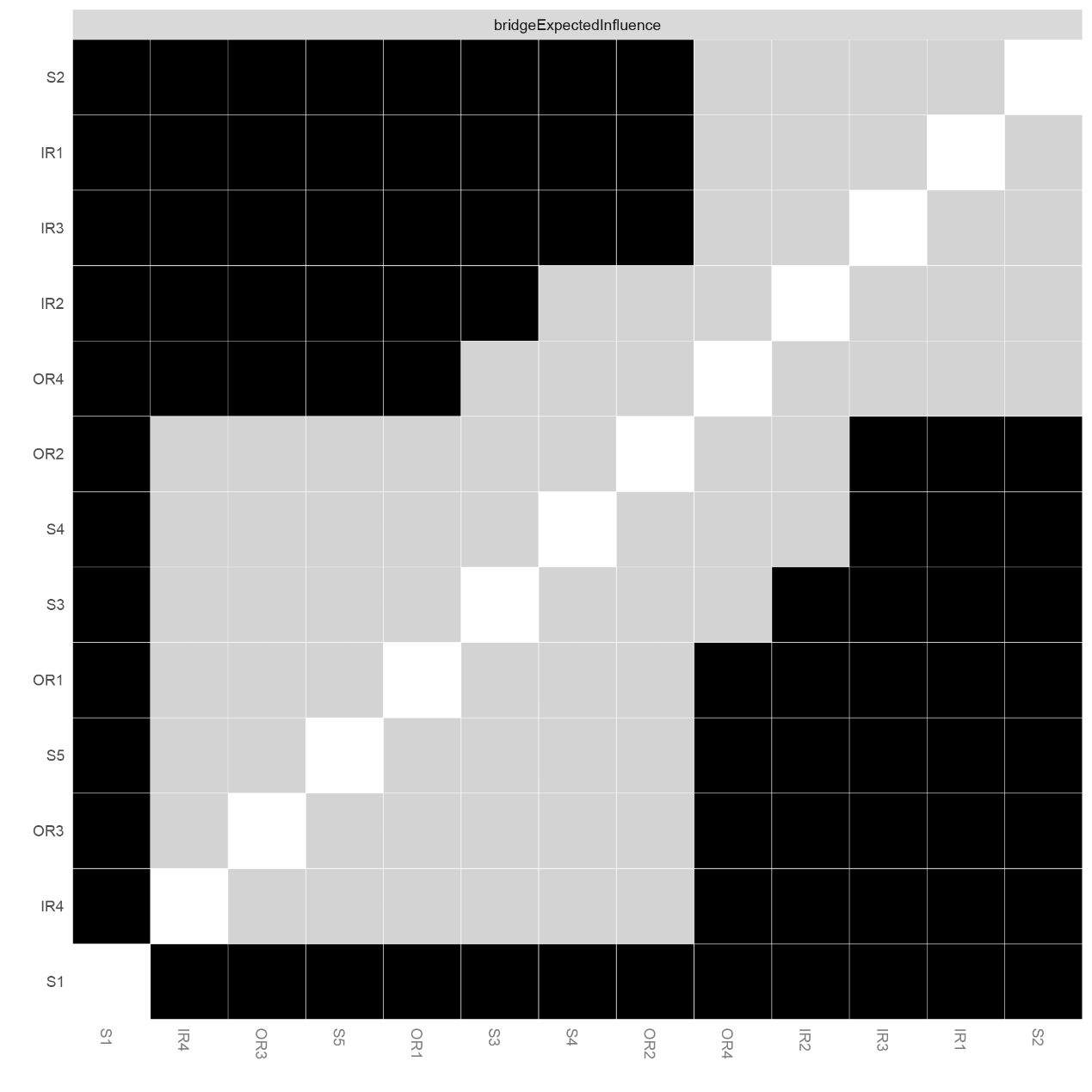


Figure S4. Bootstrapped difference test for node bridge expected influences

*Note*: Gray boxes indicate node bridge expected influences that do not differ significantly from one another, while black boxes indicate node bridge expected influences that do differ significantly.

**The results of DS network**


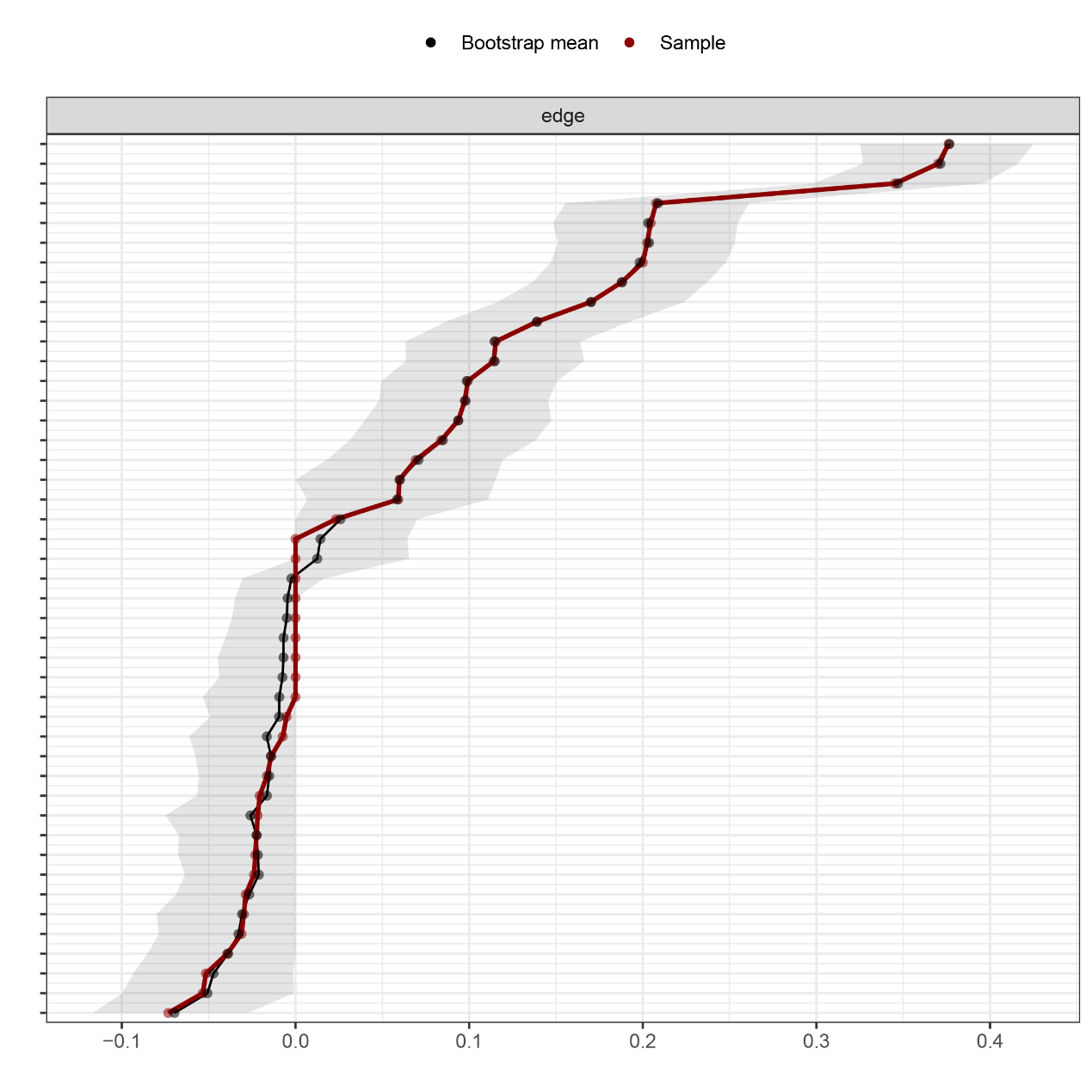


Figure S5. Accuracy of edge weights

*Note*: The red line depicts the sample edge weights and the gray bar depicts the bootstrapped confidence interval.


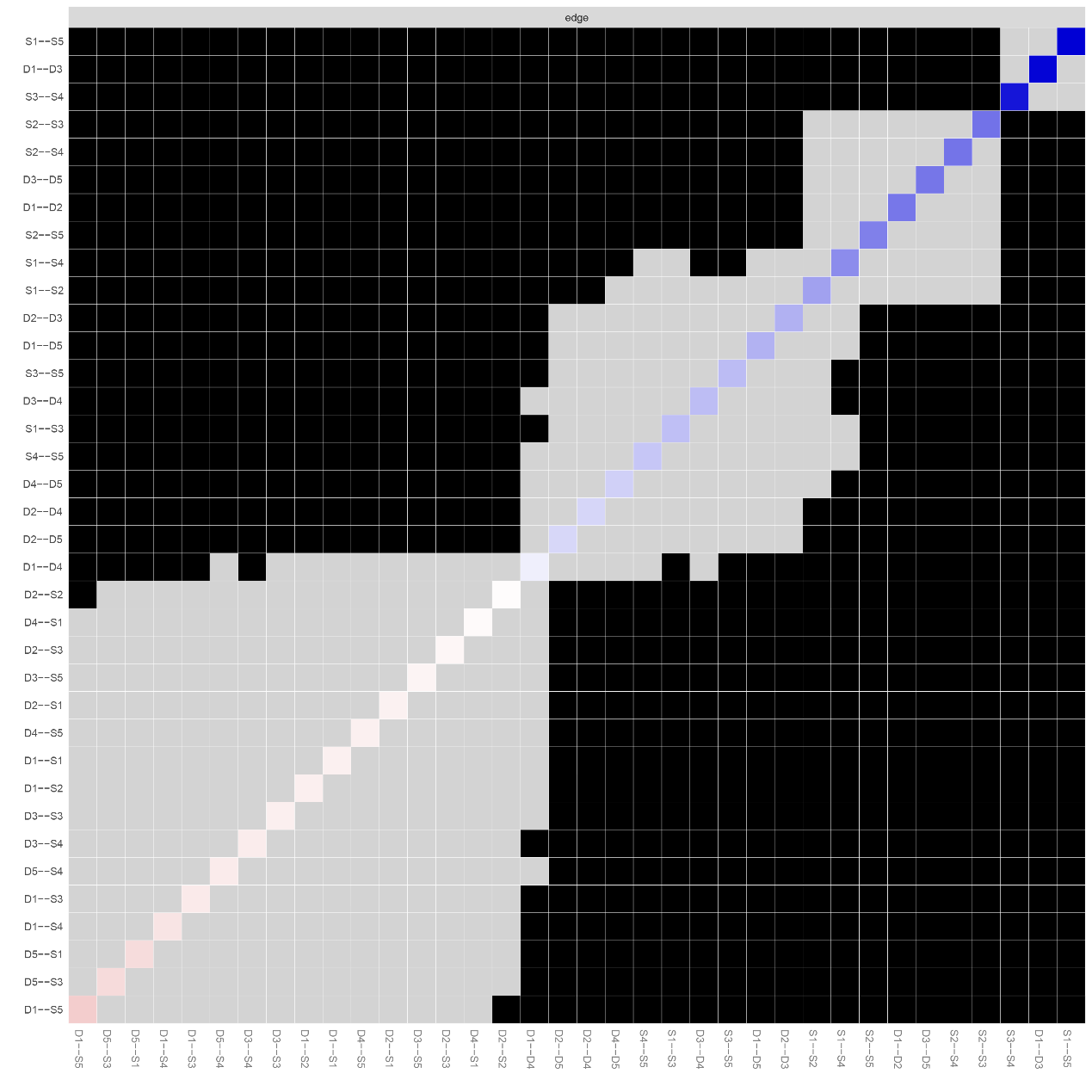


Figure S6. Bootstrapped difference test for edge weights

*Note*: Gray boxes indicate edge weights that do not differ significantly from one another, while black boxes indicate edge weights that do differ significantly. Blue and red boxes on the diagonal correspond to edge weights with positive and negative correlations, respectively.


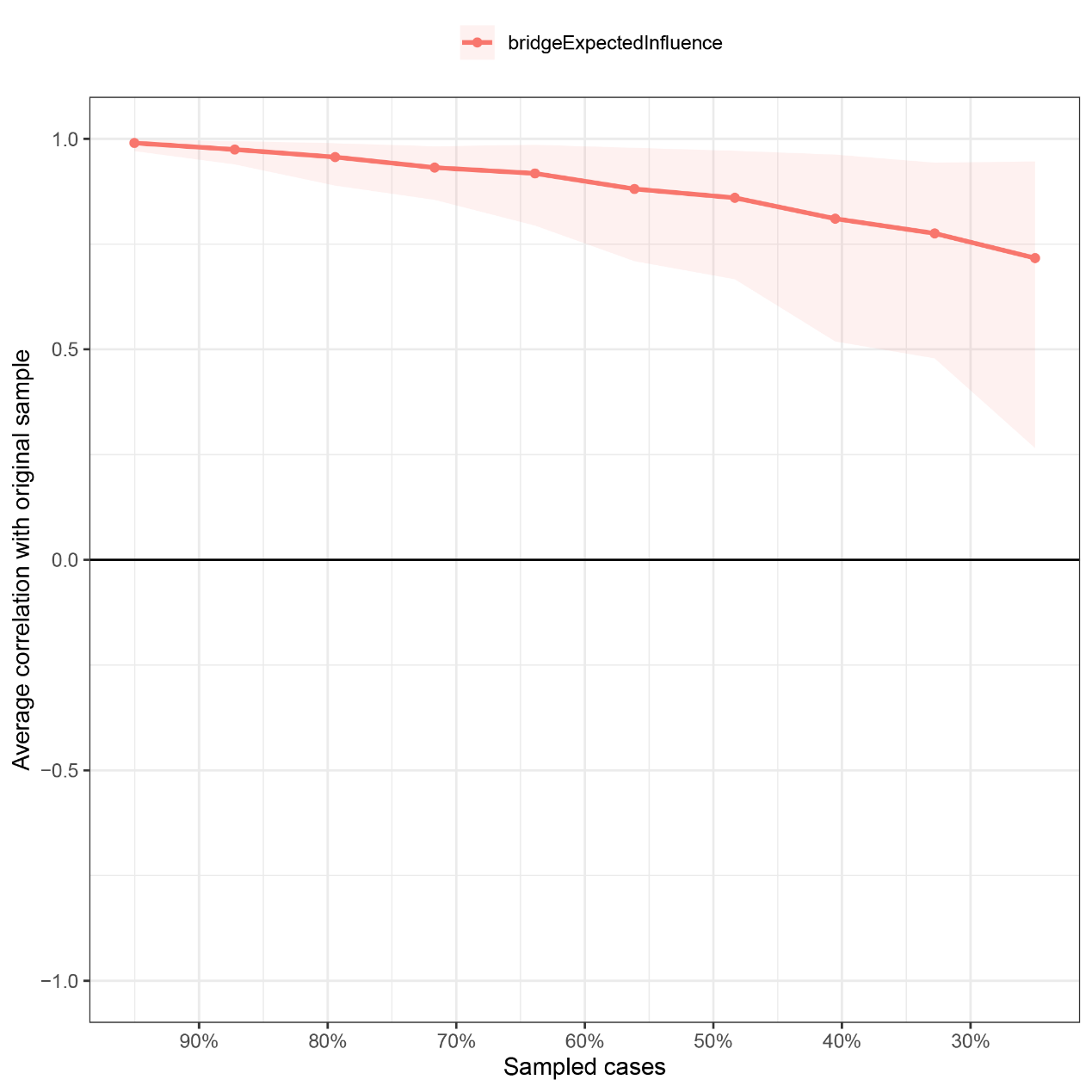


Figure S7. Stability of node bridge expected influences

*Note*: The red bar represents the average correlation between node bridge expected influences in the full sample and subsample with the red area depicting the 2.5th quantile to the 97.5th quantile.


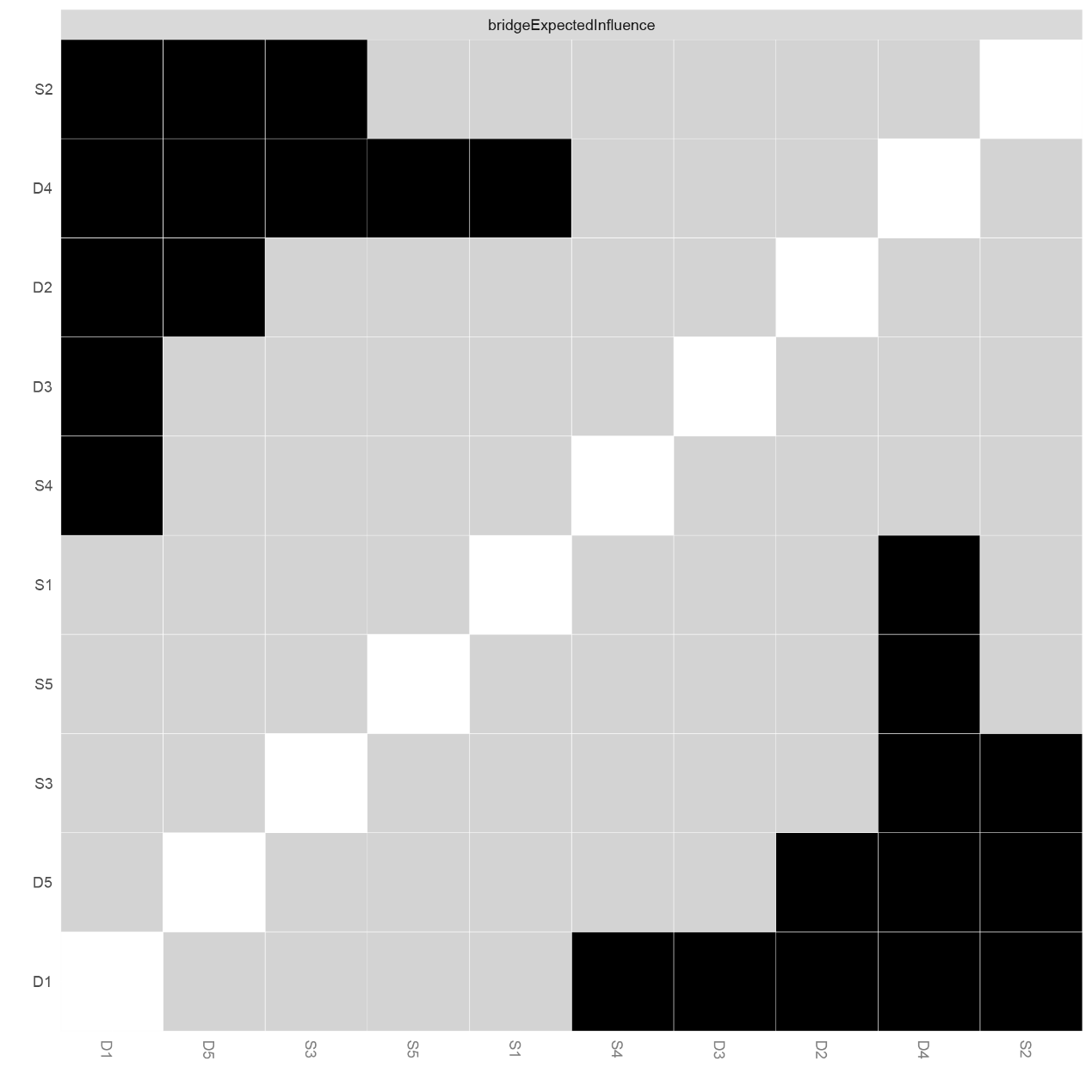


Figure S8. Bootstrapped difference test for node bridge expected influences

*Note*: Gray boxes indicate node bridge expected influences that do not differ significantly from one another, while black boxes indicate node bridge expected influences that do differ significantly.

**The results of AS network**


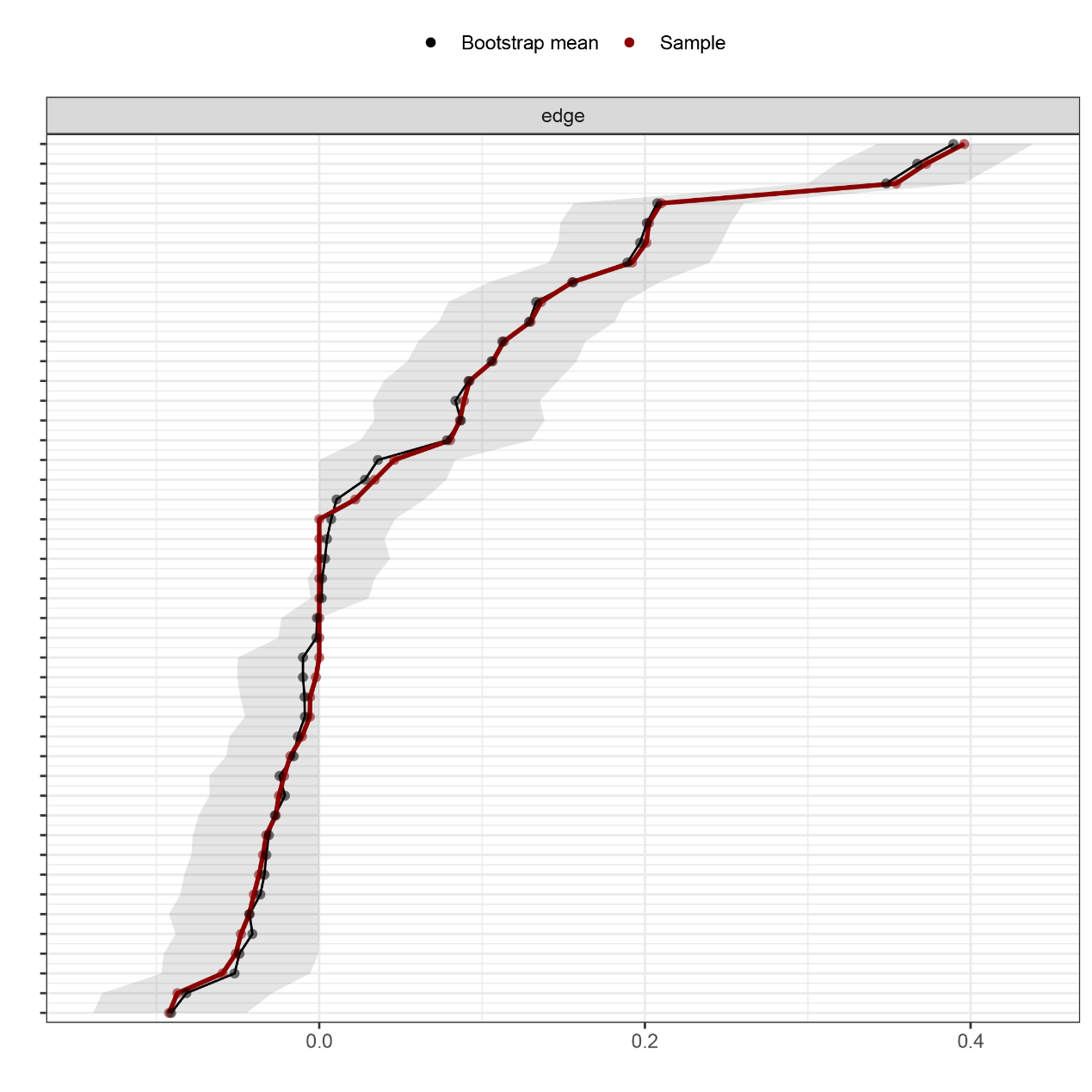


Figure S9. Accuracy of edge weights

*Note*: The red line depicts the sample edge weights and the gray bar depicts the bootstrapped confidence interval.


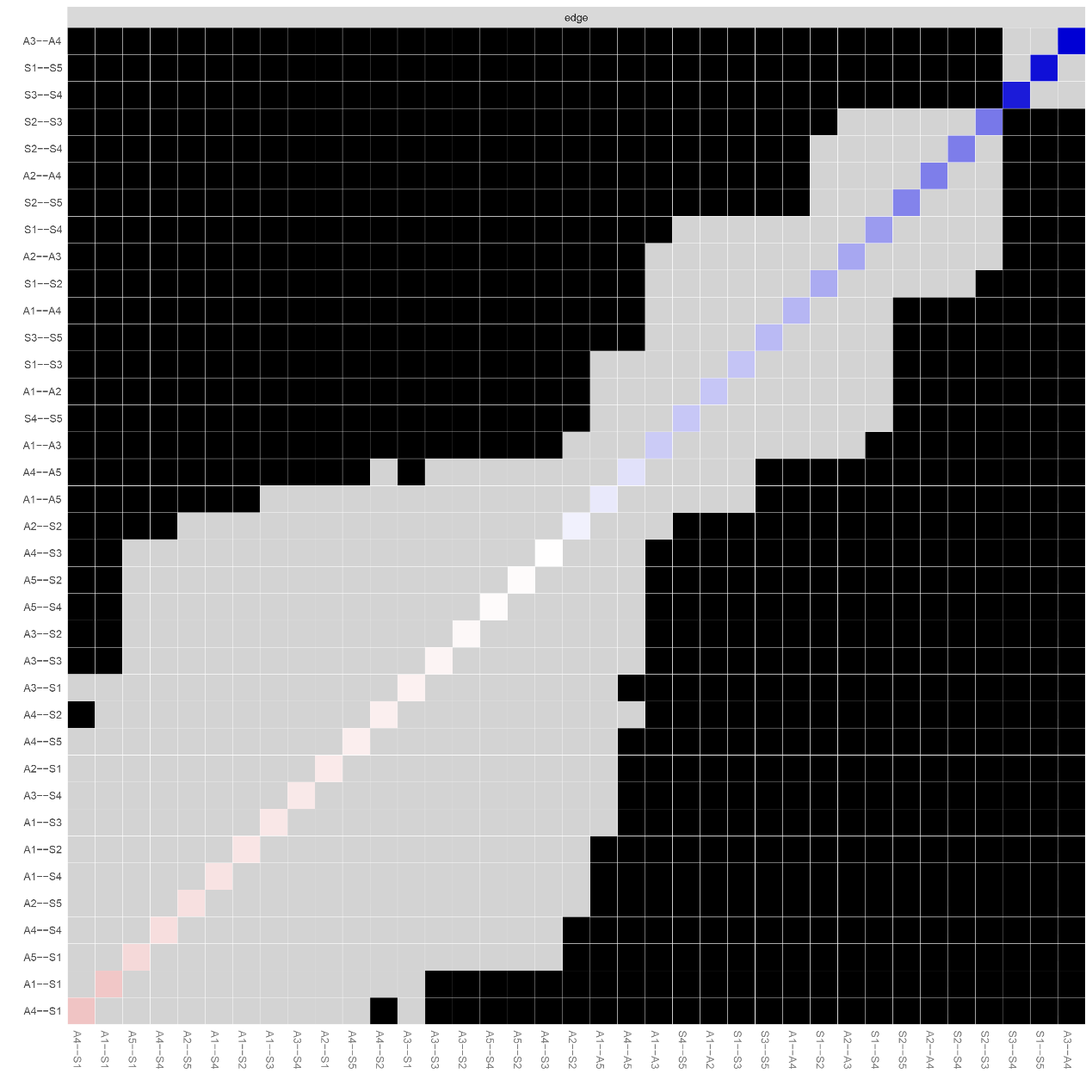


Figure S10. Bootstrapped difference test for edge weights

*Note*: Gray boxes indicate edge weights that do not differ significantly from one another, while black boxes indicate edge weights that do differ significantly. Blue and red boxes on the diagonal correspond to edge weights with positive and negative correlations, respectively.


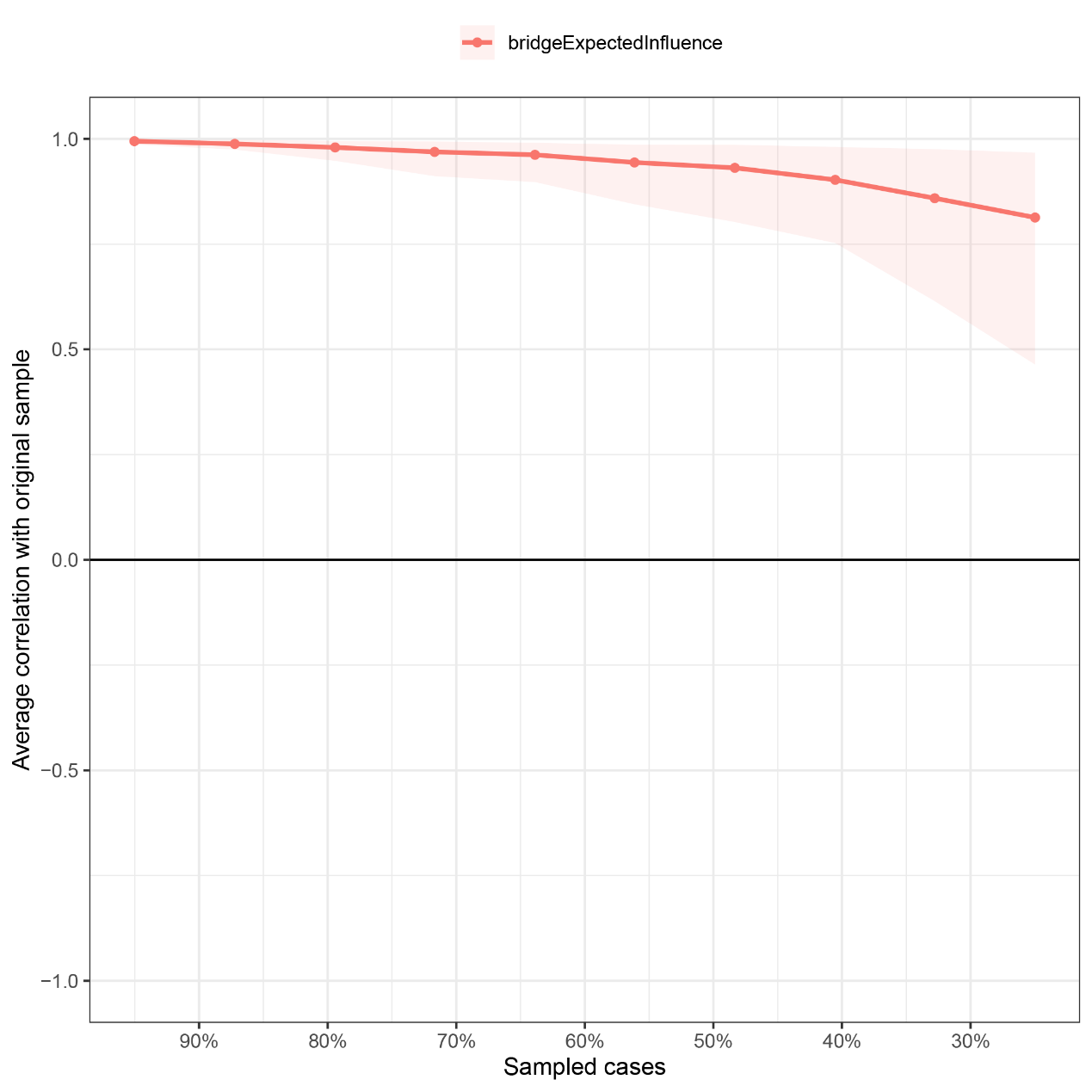


Figure S11. Stability of node bridge expected influences

*Note*: The red bar represents the average correlation between node bridge expected influences in the full sample and subsample with the red area depicting the 2.5th quantile to the 97.5th quantile.


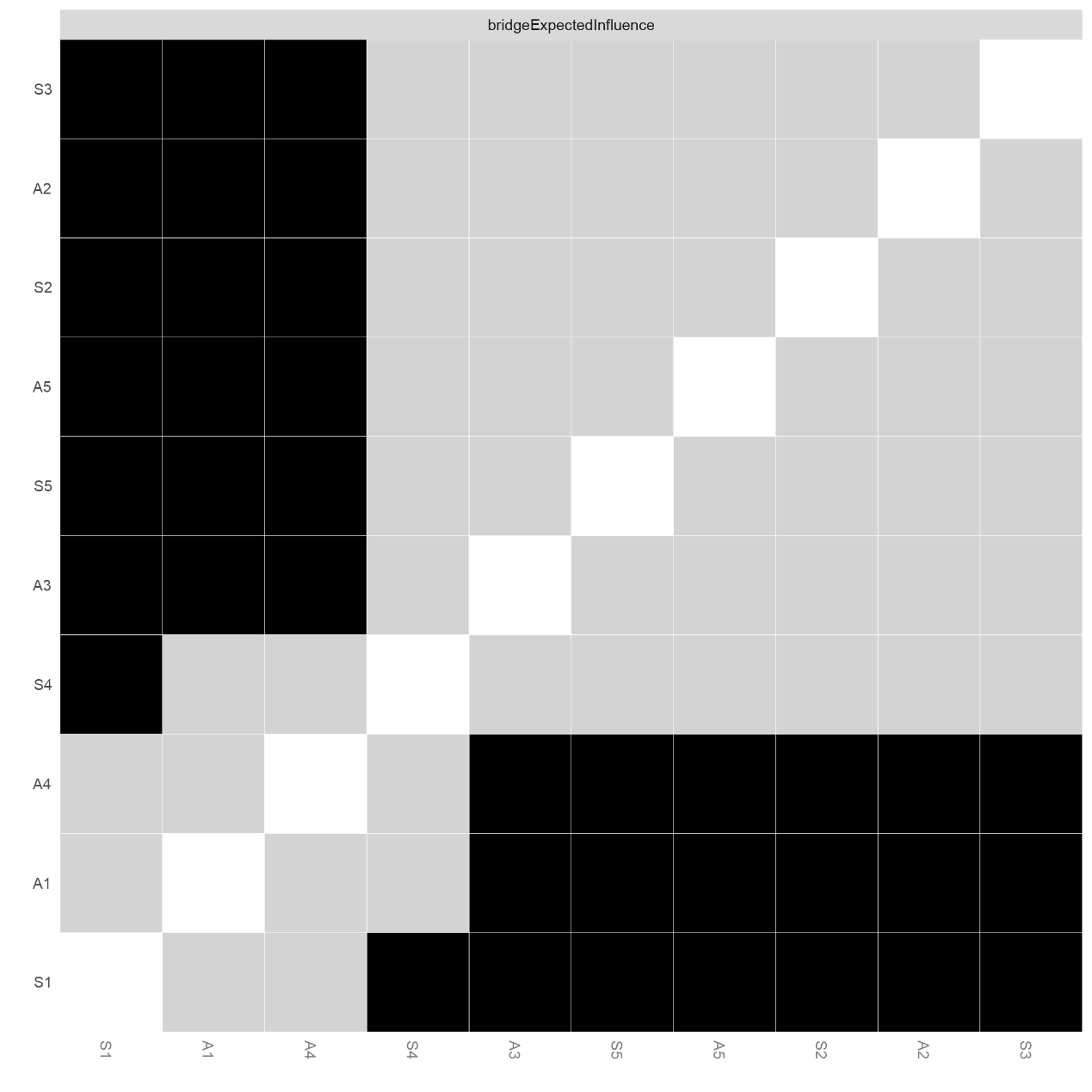


Figure S12. Bootstrapped difference test for node bridge expected influences

*Note*: Gray boxes indicate node bridge expected influences that do not differ significantly from one another, while black boxes indicate node bridge expected influences that do differ significantly.
